# Role of Ca^2+^ and L-Phe in Regulating Functional Cooperativity of Disease-Associated “Toggle” Calcium-Sensing Receptor Mutations

**DOI:** 10.1371/journal.pone.0113622

**Published:** 2014-11-24

**Authors:** Chen Zhang, Nagaraju Mulpuri, Fadil M. Hannan, M. Andrew Nesbit, Rajesh V. Thakker, Donald Hamelberg, Edward M. Brown, Jenny J. Yang

**Affiliations:** 1 Department of Chemistry, Georgia State University, Atlanta, Georgia, United States of America; 2 Center for Diagnostics and Therapeutics, Georgia State University, Atlanta, Georgia, United States of America; 3 Academic Endocrine Unit, University of Oxford, Oxford Centre for Diabetes, Endocrinology and Metabolism, Churchill Hospital, Oxford, United Kingdom; 4 Division of Endocrinology, Diabetes and Hypertension, Department of Medicine, Brigham and Women's Hospital, Boston, Massachusetts, United States of America; University of Bari Aldo Moro, Italy

## Abstract

The Ca^2+^-sensing receptor (CaSR) regulates Ca^2+^ homeostasis in the body by monitoring extracellular levels of Ca^2+^ ([Ca^2+^]_o_) and amino acids. Mutations at the hinge region of the N-terminal Venus flytrap domain (VFTD) produce either receptor inactivation (L173P, P221Q) or activation (L173F, P221L) related to hypercalcemic or hypocalcemic disorders. In this paper, we report that both L173P and P221Q markedly impair the functional positive cooperativity of the CaSR as reflected by [Ca^2+^]_o_–induced [Ca^2+^]_i_ oscillations, inositol-1-phosphate (IP_1_) accumulation and extracellular signal-regulated kinases (ERK_1/2_) activity. In contrast, L173F and P221L show enhanced responsiveness of these three functional readouts to [Ca^2+^]_o_. Further analysis of the dynamics of the VFTD mutants using computational simulation studies supports disruption in the correlated motions in the loss-of-function CaSR mutants, while these motions are enhanced in the gain-of-function mutants. Wild type (WT) CaSR was modulated by L-Phe in a heterotropic positive cooperative way, achieving an EC_50_ similar to those of the two activating mutations. The response of the inactivating P221Q mutant to [Ca^2+^]_o_ was partially rescued by L-Phe, illustrating the capacity of the L-Phe binding site to enhance the positive homotropic cooperativity of CaSR. L-Phe had no effect on the other inactivating mutant. Moreover, our results carried out both *in silico* and in intact cells indicate that residue Leu^173^, which is close to residues that are part of the L-Phe-binding pocket, exhibited impaired heterotropic cooperativity in the presence of L-Phe. Thus, Pro^221^ and Leu^173^ are important for the positive homo- and heterotropic cooperative regulation elicited by agonist binding.

## Introduction

The human calcium (Ca^2+^)-sensing receptor (CaSR) is a seven transmembrane, G protein-coupled receptor (GPCR) that is expressed at the highest levels in the parathyroid glands and kidneys [1]. The principal role of CaSR is to sense alterations of the extracellular calcium concentration ([Ca^2+^]_o_) and to maintain Ca^2+^ homeostasis by regulating parathyroid hormone (PTH) secretion as well as renal Ca^2+^ reabsorption. Like other members of family C in the GPCR superfamily, the CaSR possesses a large extracellular domain (ECD) consisting of more than 600 amino acids [2]. Ca^2+^, the principal physiological agonist for CaSR, is thought to bind to the ECD of the dimeric form of the receptor resulting in activation of phospholipase C (PLC), with an attendant accumulation of inositol 1,4,5-triphosphate (IP_3_) followed by a resultant increase in the cytosolic calcium ([Ca^2+^]_i_) concentration [3].

Fluctuations of the plasma levels of amino acids can regulate the rate of hormone synthesis and secretion as well as Ca^2+^ metabolism, among other processes [4]. CaSR is present throughout the gastrointestinal tract [5], [6]. L-amino acids, especially aromatic amino acids, are known to enhance the sensitivity of CaSR to [Ca^2+^]_o_, which could be one potential explanation for how dietary protein modulates [Ca^2+^]_o_ homeostasis in normal individuals as well as in patients with chronic renal failure [4], [7].

Mutations of the CaSR can perturb intracellular signaling events (e.g., intracellular calcium responses) and disrupt regulation of PTH secretion from the parathyroid chief cell and Ca^2+^ reabsorption in the renal tubule. Mutations that inactivate CaSR (i.e., result in loss-of-function) cause familial hypocalciuric hypercalcemia (FHH) and neonatal severe primary hyperparathyroidism (NSHPT) [8], [9]. On the other hand, activating mutations of the CaSR lead to autosomal dominant hypocalcemia with hypercalciuria (ADHH) [10]. These mutations, including the four studied here that involve the two “toggle” residues, Leu^173^ and Pro^221^, where mutations of the same residue can either activate or inactivate the CaSR, serve as a rich source of structure-function information.

Interestingly, among thirty-four identified ECD missense mutations in patients with FHH, NSHPT and ADHH, 18 were located within 10 Å of one or more of the five Ca^2+^-binding sites predicted in our previous studies [11], particularly site 1, which is proposed to be the principal site for Ca^2+^-binding during receptor activation as well as for regulating both the homotropic and heterotropic cooperativity of CaSR [12] [13]. Our group has also reported a putative L-Phe binding site composed of residues S170, Y218, L51, S272 and T145, which are located adjacent to the predicted calcium binding pocket 1 at the hinge region of the CaSR ECD [12]. The location of the L-Phe binding site is pivotal in cooperatively regulating the activity of the CaSR [12].

Our previous computational studies showed that movements of Ca^2+^-binding site 1 are dynamically correlated with those of the other predicted calcium-binding sites. Mutations in Ca^2+^-binding site 1 could, therefore, affect both the homotropic and heterotropic cooperativity of the CaSR. These results led us to hypothesize that residues 173 and 221 are essential for the functional cooperativity orchestrated by calcium and L-Phe binding, and that related disease-associated mutations are the result of alteration or perturbation of the receptor's associated molecular connectivity.

In the present studies, we show that the loss-of-function mutations, L173P and P221Q, but not the gain-of-function mutations, L173F and P221L, alter the cooperativity of the CaSR though analyzing changes in functional readouts in response to alterations in [Ca^2+^]_o_. Furthermore, L173P and P221Q disrupt the strong correlated motions among the various calcium-binding sites as demonstrated by molecular dynamics (MD) simulations. L-Phe induced positive heterotropic cooperativity of the P221Q mutant, but had only a limited effect in potentiating the functional activity of L173P due to its restricted molecular dynamical features. These *in vitro* and *in silico* results provide important insights into how Ca^2+^ and L-Phe interact at their respective binding sites in the cleft of the VFTD, which are essential for the maintenance of calcium homeostasis that is required for normal physiological function.

## Methods

### Cell culture and transfection

Human embryonic kidney cells (HEK293) (ATCC) were cultured under standard condition (5% CO_2_, 37°C) in High Glucose Dulbecco's modified Eagle's medium (DMEM) (Sigma Chemicals) containing 10% fetal bovine serum with 100 µg/ml penicillin-streptomycin. The CaSR mutations were introduced using site-directed mutagenesis (QuikChange, Stratagene, La Jolla, CA, USA) [14]. Cells were transfected with either pEGFP-N1-WT-CaSR or mutant CaSRs using Lipofectamine 2000 in reduced serum *Opti*-MEM medium following the manufacturer's instructions (Invitrogen). After 4∼6 hours, the medium was changed to High Glucose DMEM, and the cells were incubated for an additional 48 hours to increase receptor expression. Transfection efficiency and expression levels were confirmed by analyzing the fluorescence intensity of the EGFP-tagged CaSR by fluorescence microscopy (Leica, DMI600B).

### Measurement of [Ca^2+^]_i_ responses in single cells transfected with WT or mutant CaSRs

Measurement of [Ca^2+^]_i_ was carried out as described by Jiang, et al. [15]. Briefly, wild type CaSR or its mutants were transiently transfected into HEK293 cells grown on coverslips and cultured for 48 h. The cells were subsequently loaded with Fura-2 by incubation with 4 µM Fura-2 AM in 2 mL physiological saline buffer (10 mM HEPES, 140 mM NaCl, 5 mM KCl, 1.0 mM MgCl_2_, 1 mM CaCl_2_ and pH 7.4). The coverslips were mounted in a bath chamber on the stage of a Leica DM6000 fluorescence microscope. The cells were alternately illuminated with 340 or 380 nm light, and the fluorescence at an emission wavelength of 510 nm was recorded in real time as the concentration of [Ca^2+^]_o_ was increased in a stepwise manner in the presence or absence of 5 mM L-Phe. The ratio of emission fluorescence intensity from both excitation wavelengths was then monitored as a function of [Ca^2+^]_o_ and utilized as a parameter reflecting changes in [Ca^2+^]_i_ All experiments were performed at room temperature. In addition to the use of the cell population assay as a means of calculating EC_50_ for Ca^2+^_o_ or L-Phe, the signals from 30 to 60 single cells were recorded for each measurement in a single cell [Ca^2+^]_i_ assay. In the latter assay, [Ca^2+^]_i_ oscillations in individual cells were defined as three or more successive fluctuations in [Ca^2+^]_i_ from the baseline after the initial peak.

### Immunostaining and Western blotting of CaSR

pcDNA3.1-CaSRs were used in the immunostaining and Western blotting experiments. This CaSR construct contains a flag-tag between Asp^371^ and Thr^372^. For immunostaining, 48 hours after transfection, cells were fixed with 3.7% formaldehyde for 15 min at room temperature, and subsequently labeled with mouse anti-Flag monoclonal antibody (1∶3000) overnight at 4°C. The next day cells were washed with PBS and stained with goat anti-mouse Alexa488-conjugated secondary antibodies. Images were collected on a Zeiss LSM700 confocal microscope (Carl Zeiss, German). For Western blotting, cells were lysed with RIPA buffer (Millipore, MA). Protein concentrations were determined using a Bradford assay (Bio-Rad laboratories, Hercules, CA). Lysates containing 40 µg of protein were separated by 7.5% SDS-PAGE. After gel electrophoresis, the proteins were transferred to a nitrocellulose membrane, probed with anti-flag monoclonal antibody (1∶3000) in 3% milk/TBST overnight at 4°C. Blots were subsequently probed with alkaline phosphatase-conjugated secondary antibody (1∶3000) for 1 hour at room temperature. After washing, the signals were detected by standard enhanced chemiluminescence. The signals were quantitated using ImageJ.

### Measurement of inositol-1-phosphate (IP_1_) accumulation in CaSR transfected HEK293 cells

HEK293 cells were seeded in 24-well plates at 3×10^5^ cells per well in 500 µl of culture medium. After transfection with WT CaSR or its various mutants, cells were further cultured for 24 hours at 37°C. Cell monolayers were first washed with Ringer's buffer without calcium (121 mM NaCl, 2.4 mM K_2_HPO_4_, 0.4 mM KH_2_PO_4_, 10 mM HEPES, 5.5 mM glucose, 1.2 mM MgCl_2_) and then incubated for 1 hour at 37°C in stimulation buffer (140 mM NaCl, 5 mM KCl, 10 mM LiCl_2_, 0.55 mM MgCl_2_, 10 mM HEPES) containing varying concentrations of CaCl_2_. After treatment, cells were lysed for 30 min at 37°C with 50 µl of 2.5% IP_1_ ELISA Kit Lysis Reagent (CIS Bio International, Gif-sur-Yvette, France). The accumulation of IP_1_ was measured using an immunoassay based on competition between free IP_1_ and horseradish peroxidase (HRP) conjugated IP_1_ for binding to monoclonal anti-IP_1_ antibody. The results for IP_1_ were expressed as percentage inhibition of IP_1_-HRP binding  =  [1-IP_1_-HRP binding in stimulated cells/IP_1_-HRP binding in unstimulated cells] ×100. The EC_50_s of [Ca^2+^]_o_-dependent responses were calculated by fitting the [Ca^2+^]_o_ concentration-response curves with the Hill equation.

### Determination of ERK_1/2_ phosphorylation

Thirty-six hours post transfection of monolayers of HEK293 cells with CaSR or its mutants, cells were incubated in serum-free high glucose DMEM medium supplemented with 0.2% w/v BSA at 37°C overnight. On the following day, cells were first incubated with HBSS for 30 min, followed by stimulation with varying levels of CaCl_2_ (0–20 mM) with or without L-Phe (5 mM) for 10 min. At the end of the [Ca^2+^]_o_ stimulation, cells were lysed with RIPA lysis buffer (Millipore, CA, USA). In total, 150 µg aliquots of lysate protein were loaded into either a 12.5% SDS-gel or a 4%–12.5% gradient gel for PAGE and analyzed by western blotting with an anti-phospho-p44/42 ERK polyclonal antibody (Cell Signaling Technology, Beverly, MA, USA) diluted (1∶2000). A chemiluminescent method (AP Conjugate Substrate Kit) was employed to detect the phospho-(p)44/42 proteins. Quantitative analysis of the results was performed using ImageJ software (National Institutes of Health). The responses were normalized to the maximal effect observed with [Ca^2+^]_o_ alone. The EC_50_ of [Ca^2+^]_o_-dependent responses was calculated by fitting the [Ca^2+^]_o_ concentration-response curves with the Hill equation.

### Computational analysis and Molecular Dynamics (MD) simulation of CaSR

CLUSTALW was used to align the sequences of the human CaSR ECD (residues 25–530) and the mGluR1 ECD [16]. The structure of the ECD of CaSR was modeled based on the crystal structure of mGluR1 (1EWT, 1EWK and 1ISR) using SWISS-MODEL [17] and MODELLER software [18], and the potential Ca^2+^-binding sites in the CaSR ECD were predicted using MetalFinder [19], The electrostatic potentials were calculated using Pymol.

MD simulation provides an approach complementary to experiments in live cells for understanding biomolecular structure, dynamics, and function. The initial coordinates for all the simulations were taken from a 2.20 Å resolution x-ray crystal structure with PDBID 1EWK [20]. The AMBER 10 suite of programs [21] was used to carry out all of the simulations in an explicit TIP3P water model [22], using the modified version of the all-atom Cornell et. al. [23] force field and the re-optimized dihedral parameters for the peptide ω-bond [24]. An initial 2 ns simulation was performed using NOE restraint during the equilibration in order to reorient the side chains of residues in the Ca^2+^-binding site, but no restraints were used during the actual simulation. A total of three MD simulations were carried out for 50 ns each on the apo-form and the ligand-loaded forms. During the simulations, an integration time step of 0.002 ps was used to solve the Newton's equation of motion. The long-range electrostatic interactions were calculated using the Particle Mesh Ewald method, [25], and a cutoff of 9.0 Å was applied for non-bonded interactions. All bonds involving hydrogen atoms were restrained using the SHAKE algorithm [26]. The simulations were carried out at a temperature of 300 K and a pressure of 1 bar. A Langevin thermostat was used to regulate the temperature with a collision frequency of 1.0 ps-1. The trajectories were saved every 500 steps (1ps). The trajectories were analyzed using the ptraj module in AMBER 10.

### Statistics

The data are presented as means ± SE of the indicated number of experiments. One-way repeated measures analysis of variance (ANOVA), followed by the Dunnett post hoc test was performed using SPSS (SPSS Inc, Chicago, IL, USA) for multiple comparisons to compare the differences between groups without adding L-Phe. Two-way ANOVA was used for comparison among different groups in the presence of L-Phe. A P value of < 0.05 was considered to indicate a statistically significant difference.

## Results

### The disease-associated CaSR mutations alter the [Ca^2+^]_o_-triggered [Ca^2+^]_i_ oscillation pattern

**Figure 1:**
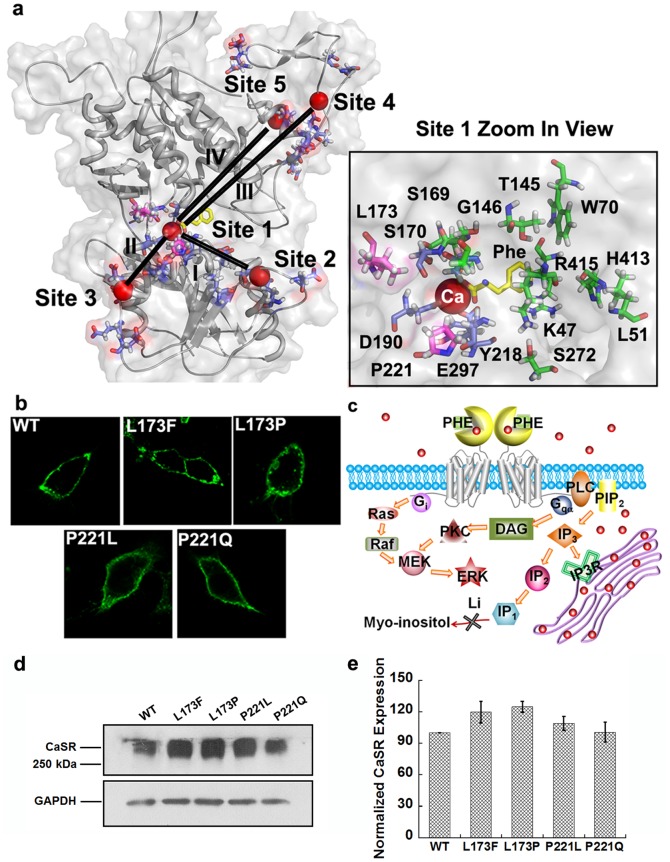
The disease-related mutations are located in the hinge region near Ca^2+^-binding site 1. a. Model structure of CaSR ECD based on the mGluR1 crystal structure (PDB entry: 1ISR) was generated using MODELER 9v4 and Swiss-Model. The global view of the ECD is shown in the left panel. Spheres highlighted in red: Ca^2+^; Purple: residues involved in predicted Ca^2+^-binding sites. L-phenylalanine (in yellow) is positioned at the hinge region between the two lobes by Autodock vina. Right panel: A zoomed in view of the Ca^2+^-binding pocket of site 1. Residues involved in Ca^2+^-binding site 1are highlighted in purple; residues with disease-related mutations are highlighted in pink; residues predicted to interact with L-Phe are presented in green. b. Immunofluorescence analysis of surface expressed WT CaSR and its mutants in HEK293 cells. Immunostaining was done with anti-flag monoclonal antibody, and detection was carried out with Alex Fluor 488-conjugated, goat anti-mouse secondary antibody. Green: CaSR. The images were taken using equal exposure times. c. Schematic figure of calcium and L-Phe induced downstream signaling changes and the principle for the measurement of IP_1_ accumulation. Red dots represent calcium ions. d. total proteins (40 µg) were applied to SDS-PAGE under non-reducing conditions and blotted with anti-flag antibody. GAPDH was used as an internal control. e. The signals were analyzed using ImageJ and the normalized intensities were compared with WT CaSR.

The two residues (Leu^173^, Pro^221^) involved in the four disease-associated mutations studied here are located in the hinge region near the predicted calcium-binding site 1, which is located between lobe 1 and lobe 2. Meanwhile they are also adjacent (within 10 Å) to the reported L-Phe binding pocket formed by residues K47, L51, W70, T145, G146, S169, S170, I187, Y218, S272, H413 and R415, which can exert effects over a large region of the ECD through positive heterotropic cooperativity as shown in Figure 1a [12]. The immunostaining as well as the Western blotting results suggest that all of the CaSR mutants are expressed at the cell surface at similar levels (Figure 1b, 1d, 1e).

**Figure 2:**
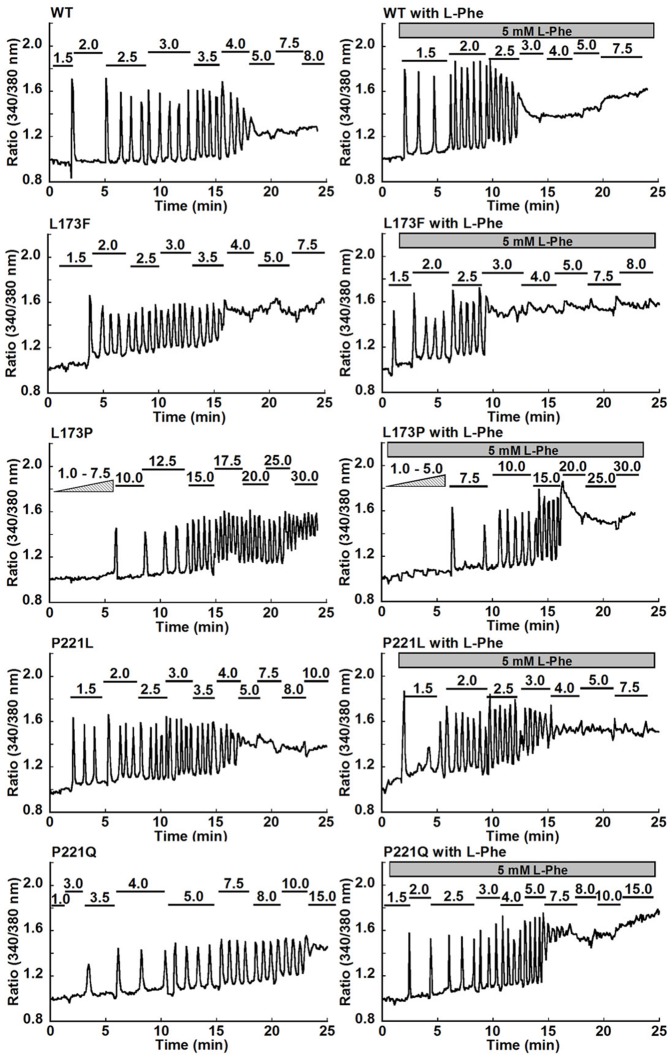
Functional studies of disease-related CaSR mutations in individual HEK293 cells. The various panels show representative oscillation patterns from single cells. HEK-293 cells transfected with CaSR or one of its mutants were loaded with Fura-2 AM for 15 min. [Ca^2+^]_i_ was assessed by monitoring emission at 510 nm with excitation alternately at 340 or 380 nm as described in Methods. Each experiment was carried out with or without 5 mM L-Phe and began in Ca^2+^-free Ringer's buffer (10 mM HEPES, 140 mM NaCl, 5 mM KCl, and 1.0 mM MgCl_2_, pH 7.4), followed by stepwise increases in [Ca^2+^]_o_ until [Ca^2+^]_i_ reached a plateau (up to 30 mM [Ca^2+^]_o_).

**Figure 3:**
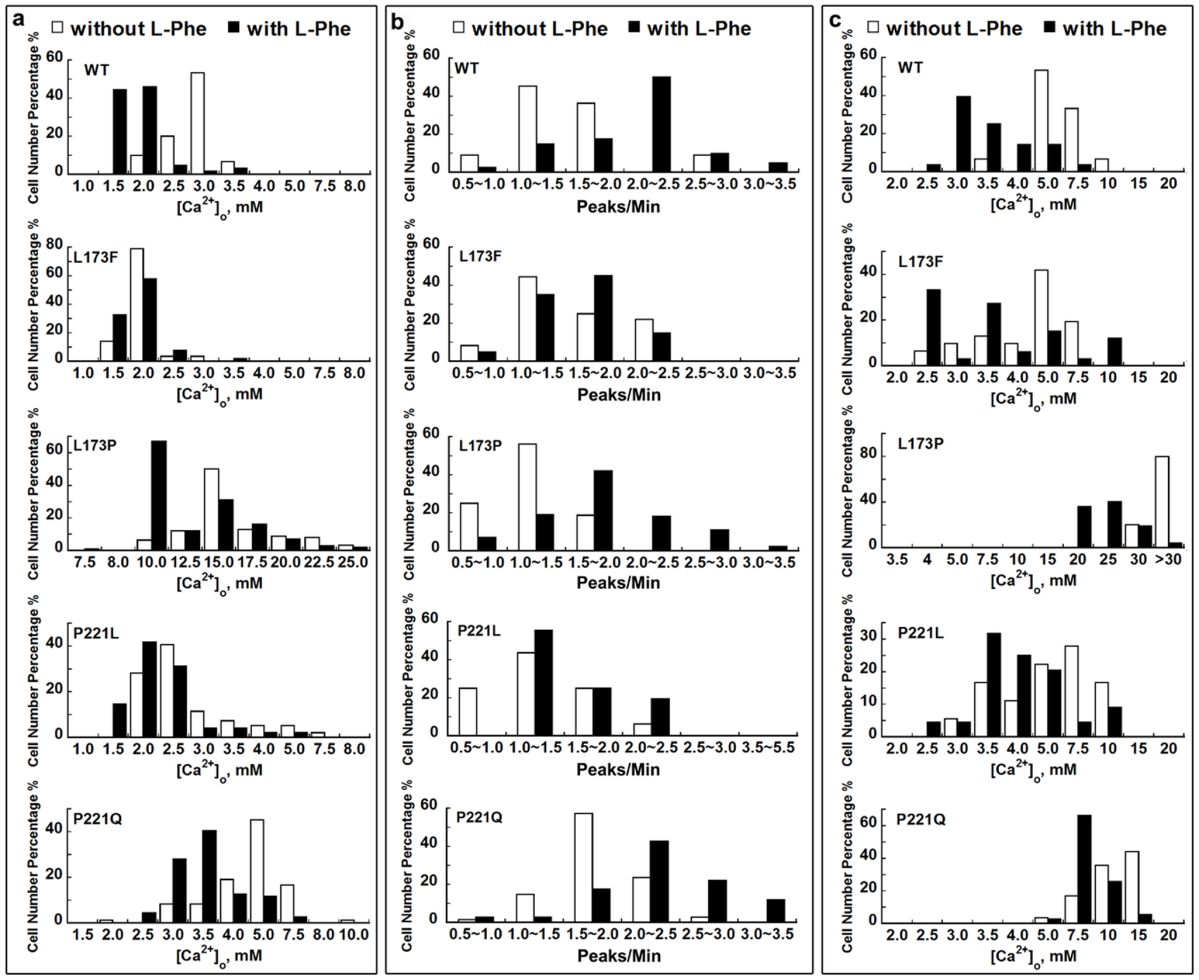
Frequency distribution of oscillation parameters in HEK293 cells transfected with CaSR or its mutants. The pattern of the [Ca^2+^]_i_ response in each cell (minimum of 40 cells) was analyzed. a. The [Ca^2+^]_o_ at which individual cells started to oscillate was recorded. The X-axis comprises 1.0 mM to 8.0 mM [Ca^2+^]_o_ for WT and for mutants L173F and P221L, 1.5 mM to 10.0 mM [Ca^2+^]_o_ for P221Q or 7.5 mM to 25.0 mM for mutant L173P. b. The frequency of the individual cell oscillation patterns was investigated. For experiments without L-Phe, the peaks per minute were recorded at the levels of [Ca^2+^]_o_ at which the majority of the cells (>50%) started oscillating. Specifically, for the gain-of-function mutants, the peaks per minute were recorded at 2.5 mM [Ca^2+^]_o_, while for loss-of-function mutants, the frequency was analyzed at 15.0 mM [Ca^2+^]_o_ for L173P and 5.0 mM [Ca^2+^]_o_ for P221Q. c. The [Ca^2+^]_o_ at which the [Ca^2+^]_i_ oscillations began to reach a plateau was recorded. Empty bar: in the absence of L-Phe; Black bar: in the presence of 5 mM L-Phe.

Analysis of the [Ca^2+^]_o_-triggered [Ca^2+^]_i_ signaling (Figure 1c), especially the pattern of [Ca^2+^]_i_ oscillations, is a straightforward method to depict the potential alterations in the homotropic cooperativity induced by mutations. Three parameters were employed to analyze the [Ca^2+^]_i_ oscillation patterns: the starting point, which refers to the [Ca^2+^]_o_ at which a given cell starts to show at least three continuous, sequential peaks; the frequency, which is defined as the number of peaks of [Ca^2+^]_i_ per minute, and the ending point, which is the [Ca^2+^]_o_ at which the [Ca^2+^]_i_ ceases to oscillate and reaches a plateau. About 53% of the cells transfected with WT CaSR showing oscillatory responses started to oscillate at 3.0 mM [Ca^2+^]_o_ (Figure 2 & 3). However, the majority of cells transfected with the inactive mutant, P221Q, did not oscillate until [Ca^2+^]_o_ reached more than 4.0 mM, and the threshold for [Ca^2+^]_i_ oscillations in cells transfected with the other inactive mutant, L173P, was even higher at 12.5 mM. Conversely, for the gain-of-function mutations (L173F and P221L), the majority of the cells, 79% for L173F and 40% for P221L, started to oscillate at lower levels of [Ca^2+^]_o_, 2.0 mM and 2.5 mM, respectively.

WT CaSR-transfected cells exhibited a normal distribution of oscillation frequencies with an average frequency of 1.0∼1.5 peaks/min at 3.0 mM [Ca^2+^]_o_ (Figs. 2&3b, Table 1). The two inactivating mutants (L173P and P221Q) responded to changes in [Ca^2+^]_o_ over a substantially different range. Therefore, the frequencies were measured at the level of [Ca^2+^]_o_ at which the majority of the cells (>50%) started to oscillate—15 mM for L173P and 5 mM for P221Q. For L173F and P221L, the frequencies were measured at 2.5 mM [Ca^2+^]_o_. The oscillation frequency for the two inactivating mutants did not differ from that of the WT (1.3±0.1 peaks/min) and were 1.3±0.1 peaks/min for L173P and 1.5±0.1 peaks/min for P221Q, but there were slight but significant increases for L173F (1.7±0.1 peaks/min) and P221L (1.6±0.1 peaks/min).

**Table 1:** Summary of cellular responses of HEK293 cells transiently transfected with WT CaSR or disease related mutants.

Mutants	EC_50_[Ca^2+^]_O_		Hill coefficient		Frequency	
	w/o L-Phe	with 5 mM L-Phe	w/o L-Phe	with 5 mM L-Phe	w/o L-Phe	with 5 mM L-Phe
**WT**	3.0±0.2	1.9±0.3*	3.7±0.3	5.0±0.7*	1.3±0.1	2.1±0.1*
**L173F**	1.9±0.2^#^	1.6±0.1	3.3±0.1	3.7±0.3	1.7±0.1^#^	1.9±0.1
**P221L**	2.0±0.1^#^	1.7±0.1	3.2±0.2	2.8±0.2	1.6±0.1^#^	1.8±0.2
**L173P (Phase 1)**	3.8±0.3	3.1±0.2	3.3±0.6	2.5±0.2	1.3±0.1	1.8±0.2*
**L173P (Phase 2)**	13.0±0.3	12.2±0.2*	3.6±0.4	5.8±0.3		
**P221Q**	5.2±0.4^#^	3.7±0.4*	2.4±0.4^#^	4.1±0.7*	1.5±0.1	2.2±0.1*

The average levels of [Ca^2+^]_o_ at which cells started to exhibit [Ca^2+^]_i_ oscillations were recorded for WT or each mutant CaSR. For the oscillation frequencies in the absence of L-Phe, peaks per minute were measured at the level of [Ca^2+^]_o_ at which more than 50% cells started to oscillate; when L-Phe was added, frequencies were recorded at the same [Ca^2+^]_o_ as their counterparts without L-Phe. Specifically, the frequencies of WT was measured at 3.0 mM [Ca^2+^]_o_, at 2.5 mM [Ca^2+^]_o_ for L173F and P221L; at 15.0 mM [Ca^2+^]_o_ for L173Pand at 5.0 mM [Ca^2+^]_o_ for P221Q. Curve-fitting was performed using the Hill equation. The data were obtained from three experiments for each construct. Values are means ± S.E. EC_50_ and Hill numbers obtained from the cell population assay by fitting plots using the Hill equation. ^#^ indicates significance with respect to wild type CaSR in the absence of L-Phe, p<0.05 (ANOVA, Dunnett test); ^*^ indicates significance with respect to the corresponding mutants in the absence of L-Phe, p<0.05 (two-way ANOVA).

We also studied the oscillation ending point. About 50% of the oscillatory cells expressing the CaSR reached a [Ca^2+^]_i_ plateau (i.e., stopped oscillating) at 5.0 mM [Ca^2+^]_o_ (Figure 3c). The loss-of-function mutations exhibited a dramatic increase in the [Ca^2+^]_o_ required for a [Ca^2+^]_i_ plateau as shown in Figure 3c. The majority of the oscillatory cells transfected with L173P were still exhibiting [Ca^2+^]_i_ fluctuations even at a level of [Ca^2+^]_o_ of 30.0 mM. Similarly, the ending point for P221Q was substantially elevated at ∼10.0–15.0 mM. On the other hand, quite a few cells transfected with either of the gain-of-function mutants stopped [Ca^2+^]_i_ oscillations at a [Ca^2+^]_o_ below 5.0 mM. Therefore, the [Ca^2+^]_o_-triggered [Ca^2+^]_i_ oscillation pattern, including its frequency, the [Ca^2+^]_o_ required for initiating oscillations, and the oscillation ending point are altered in the four mutations, suggesting changes in [Ca^2+^]_o_–elicited positive homotropic cooperativity induced by these mutations.

#### The disease-associated CaSR mutations affect functional positive homotropic cooperativity as reflected by changes in multiple CaSR-activated signaling pathways

**Figure 4:**
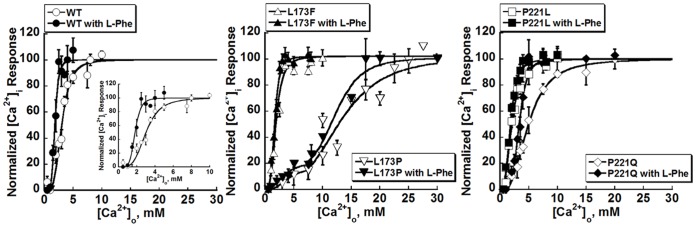
L-Phe modulates the [Ca^2+^]_o_ concentration response curves in CaSR-transfected HEK293 cells. The [Ca^2+^]_i_ responses of HEK293 cells transiently overexpressing WT CaSR or disease-related mutations were measured using Fura-2AM during stepwise increases in [Ca^2+^]_o_ from 0.5 to 30 mM with or without L-Phe as above. The ratio of light emitted at 510 nm upon excitation alternately with 340 or 380 nm was normalized to the maximum response. And the average [Ca^2+^]_i_ responses at various [Ca^2+^]_o_ were normalized and plotted against [Ca^2+^]_o_ and further fitted using the Hill equation. Open marker: in the absence of L-Phe; Closed marker: in the presence of 5 mM L-Phe.

We then investigated how those mutations affect the CaSR-mediated activation of various intracellular signaling pathways. HEK293 cells transfected with WT CaSR exhibited sigmoidal concentration response curves for the [Ca^2+^]_i_ responses to increases in [Ca^2+^]_o_ in a cell population assay with a Hill coefficient of 3.7±0.3 and an EC_50_ of 3.0±0.2 mM, suggesting strong positive homotropic cooperativity among its five predicted Ca^2+^-binding sites (Figure 4, Table 1). Notably, the two inactivating mutations showed increases in EC_50_ and disruption of the CaSR's positive homotropic cooperativity, with a change in the monophasic [Ca^2+^]_o_-induced [Ca^2+^]_i_ response curves to a biphasic one (L173P) or a decrease in Hill number for P221Q, accompanied by an increase of EC_50_ to 5.2±0.4 mM (Figure 4, Table 1).

**Figure 5:**
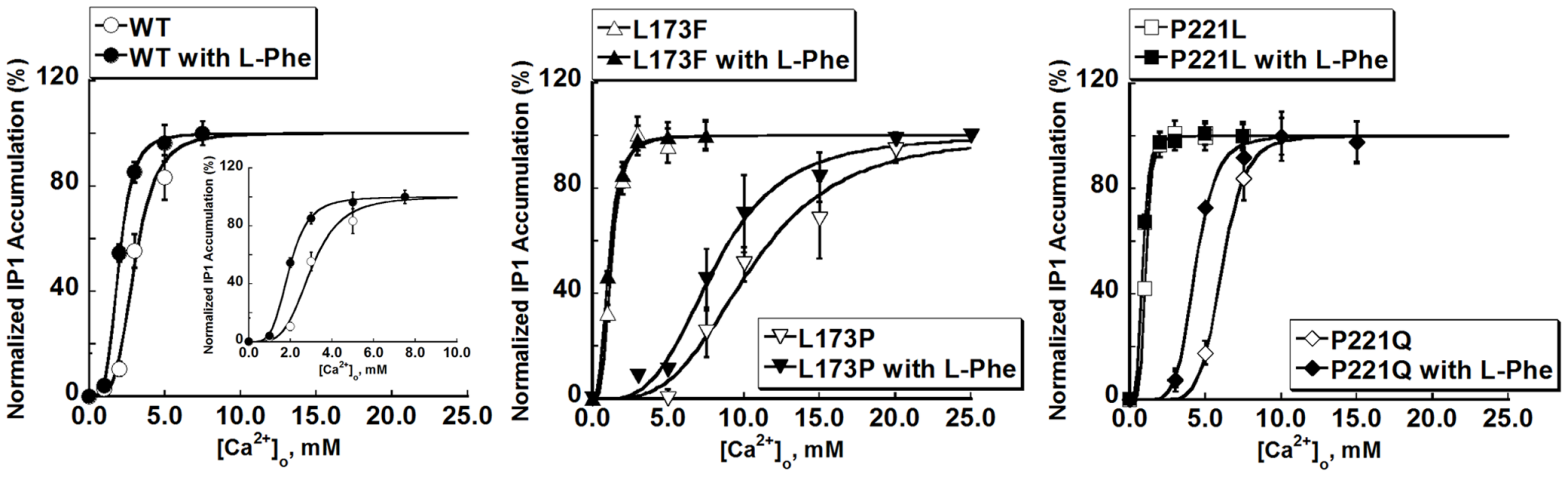
L-Phenylalanine potentiates [Ca^2+^]_o_-induced IP_1_ accumulation. IP_1_ accumulation was measured using the IP-One ELISA Kit as detailed in Methods. Left: Changes in IP_1_ accumulation were measured in response to incubation with 0, 1.0, 2.0, 3.0, 5.0 and 7.5 mM [Ca^2+^]_o_ in HEK293 cells transfected with WT CaSR, or with mutants L173F or P221L. For mutant L173P, IP_1_ accumulation was measured in response to 0, 3.0, 5.0, 7.5, 10.0, 15.0, 20.0 and 25.0 mM [Ca^2+^]_o_; for mutant P221Q, the IP_1_ responses at 0, 3.0, 5.0, 7.5, 10.0, 15.0 mM [Ca^2+^]_o_ were recorded. The IP_1_ response-calcium concentration responses were fitted using Hill equation as described in Methods.

Activation of the G_q/11_ pathway by GPCRs activates phospholipase C (PLC), which induces an increase in [Ca^2+^]_i_ caused by the associated accumulation of inositol phosphates (IPs) [3]. Thus the activation of PLC in response to increases in [Ca^2+^]_o_ in cells transiently transfected with WT CaSR were assessed by measuring the accumulation of IP_1_ as an index of PLC activation Figure 1c & Figure 5). The EC_50_ for the stimulation of IP_1_ accumulation was 2.9±0.3 mM, similar to the EC_50_ calculated from the [Ca^2+^]_i_ responses (3.0±0.2). As with their Ca^2+^_i_ responses, L173P and P221Q exhibited impaired [Ca^2+^]_o_–stimulated IP_1_ accumulation as reflected by the increases in their EC_50_s, 10.7±1.0 mM and 6.1±0.3 mM respectively, vs. 2.9±0.3 mM for the WT) (Figure 5, Table 2). In contrast, the L173F and P221L mutants showed substantial decreases in their EC_50_s to 1.2±0.1 mM and 1.1±0.1 mM, respectively (Table 2).

**Table 2:** Summary of EC_50_s from experiments measuring IP_1_ accumulation and ERK_1/2_ phosphorylation.

Mutants	EC_50_ (IP_1_-Elisa)		EC_50_ (ERK_1/2_ activity)	
	Without L-Phe With L-Phe	With L-Phe	Without L-Phe	With L-Phe
**WT**	2.9±0.3	2.0±0.1*	3.0±0.1	1.9±0.1*
**L173F**	1.2±0.1^#^	1.0±0.1	2.7±0.1^#^	1.6±0.1*
**P221L**	1.1±0.1^#^	0.9±0.1	2.2±0.3^#^	1.7±0.2
**L173P**	10.7±1.0^#^	9.0±0.9	14.9±1.0^#^	13.5±0.8
**P221Q**	6.1±0.3^#^	4.5±0.4*	11.9±0.3^#^	9.5±0.4*

HEK293 cells were transiently transfected with the WT CaSR or disease-associated CaSR mutants. Cells were then treated with various levels of [Ca^2+^]_o_. The EC_50_s of [Ca^2+^]_o_-IP_1_ responses and the -[Ca^2+^]_o_-ERK_1/2_ phosphorylation activity responses were obtained from curve fitting using the Hill equation as mentioned in Method. ^#^ indicates significance with respect to wild type CaSR, p<0.05 (ANOVA, Dunnett test); ^*^ indicates significance with respect to the corresponding mutants in the absence of L-Phe, p<0.05 (two-way ANOVA).

**Figure 6:**
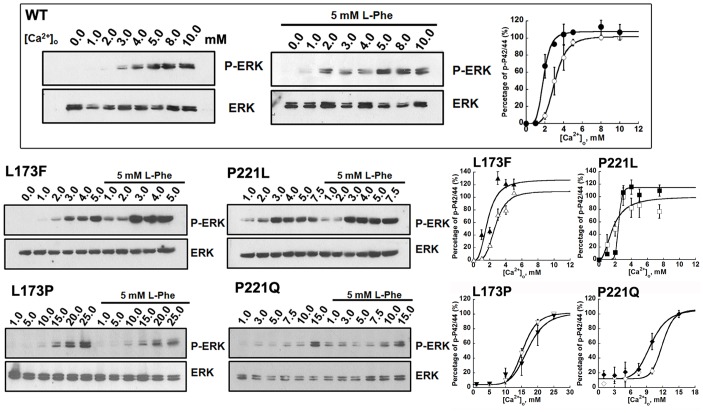
L-Phe potentiates [Ca^2+^]_o_-activated ERK signaling in CaSR-transfected HEK293 cells. HEK-293 cells transfected with WT CaSR or its mutants were incubated in serum-free high glucose MEM medium containing 0.2% BSA overnight. Cells were washed with Hank's balance salt solution (HBSS) and then incubated in the presence of various Ca^2+^ concentrations (0.0-∼25.0mM) in the absence or presence of 5 mM L-phenylalanine for 10 min at 37°C. The incubations were stopped by exposure to the lysis buffer and processed for SDS/PAGE and Western blotting as described in the Methods. The Western blot results were further quantified using Image J. All [Ca^2+^]_o_-concentration response curves were normalized to the maximum response in each individual experiment. The Hill equation was employed to fit the data. Markers: [Ca^2+^]_o_ only; closed markers: with L-Phe.

We further examined the effects of [Ca^2+^]_o_ on mitogen-activated protein kinases (MAPK) (ERK_1/2_) activation. Exposure of WT CaSR-transfected HEK293 cells to increasing concentrations of [Ca^2+^]_o_ in the range of 0.0-∼2.0 mM for 10 minutes had little effect on the phosphorylation of p44/42 ERK. Greater increases of [Ca^2+^]_o_ resulted in the accumulation of p44/42 ERK, which exhibited a maximum response at 8.0 mM [Ca^2+^]_o_. Immunoblots of [Ca^2+^]_o_–induced increases in ERK activity were further quantified using ImageJ and plotted against [Ca^2+^]_o_. The data were then fitted using the Hill equation, giving an EC_50_ of around 3.0±0.1 mM, which is close to the result measured using intracellular calcium responses. The mutants L173P and P221Q also impaired the [Ca^2+^]_o_-triggered ERK activity of the CaSR. More than 8.0 mM [Ca^2+^]_o_ was needed in order to induce the phosphorylation of ERK_1/2_ for both mutants (Figure 6). For the activating mutation, L173F, a phosphorylated ERK_1/2_ signal could be detected at 2.0 mM [Ca^2+^]_o_, which was in line with its enhanced intracellular calcium response compared to WT CaSR. The mutant P221L also potentiated [Ca^2+^]_o_-evoked ERK_1/2_ activity in the transfected cells, approaching a maximum at 3.0 mM [Ca^2+^]_o_.

#### Disease-associated mutations affect the heterotropic positive cooperativity induced by L-Phe on the CaSR

We assessed the impact of L-Phe on [Ca^2+^]_o_–induced changes in [Ca^2+^]_i_ in HEK293 cells transfected with WT CaSR in our recent work [12], however, it was not clear how the disease-associated mutations would influence the heterotropic positive cooperativity generated by L-Phe. For the majority of WT CaSR-transfected HEK293 cells, [Ca^2+^]_i_ oscillations first began at 1.5 mM [Ca^2+^]_o_ and ended at 3.0 mM [Ca^2+^]_o_ in the presence of 5 mM L-Phe, and the oscillation frequency at 3.0 mM [Ca^2+^]_o_ increased to 2.1±0.1 peaks/min, which was significantly higher than the 1.3±0.1 peaks/min observed in the absence of L-Phe (Figure 3). For the loss-of-function mutants, cells transfected with mutant L173P started oscillating at 10.0 mM [Ca^2+^]_o_ with 5.0 mM L-Phe (Figure 3). L-Phe increased the frequency of the L173P mutant significantly from 1.3±0.1 to 1.8±0.2 peaks/min at 15.0 mM [Ca^2+^]_o_ in the presence of L-Phe (Figure 3b & Table 1). In most cells, the [Ca^2+^]_i_ oscillations stopped at 25.0 mM [Ca^2+^]_o_ (Figure 3c). In contrast, without L-Phe, 80% of the cells were still oscillating at 30 mM [Ca^2+^]_o_. For the P221Q mutant, addition of 5 mM L-Phe at 5.0 mM [Ca^2+^]_o_ decreased the threshold for initiation of [Ca^2+^]_i_ oscillations from 5.0 mM to 3.5 mM [Ca^2+^]_o_, increased the oscillation frequency from 1.5±0.1 peaks/min to 2.2±0.1 peaks/min, and reduced the [Ca^2+^]_o_ required to reach the ending point. Regarding the gain-of-function mutations (L173F, P221L), L-Phe lowered the threshold for the termination of [Ca^2+^]_i_ oscillations, but barely shifted the frequency distribution of their starting points nor significantly increased their frequencies at 2.5 mM [Ca^2+^]_o_ (Figs. 2 & 3).

Meanwhile, L-Phe facilitated the response of the WT CaSR to [Ca^2+^]_o_ by significantly decreasing the EC_50_ for [Ca^2+^]_i_ responses from 3.0±0.2 mM to 1.9±0.3 mM (p<0.05, two-way ANOVA) and increasing the Hill coefficient to 5.0±0.7 mM (Figure 4). Similar analyses were carried out for the four CaSR mutants. L-Phe shifted the sigmoidal curve to the left producing a lower EC_50_ (3.7±0.3 mM) with an increase in Hill number (4.1±0.7 mM) for mutant P221Q as measured from the [Ca^2+^]_o_-evoked increase in [Ca^2+^]_i_. L-Phe did not left-shift the activation of the L173P mutant by [Ca^2+^]_o_ to the same extent that it did with the other loss-of-function mutant, P221Q, potentially because of the proximity of this residue to the binding site for L-Phe, which might interfere with L-Phe binding. The disruption of the positive homotropic cooperativity induced by [Ca^2+^]_o_ as a result of the Leu to Pro change, which was reflected as a biphasic concentration response curve, was also not corrected by the addition of L-Phe (Figure 4). On the other hand, the addition of L-Phe barely induced any further left-shift in the concentration-response curves of the two gain-of-function CaSR mutants (p>0.05).

The influence of the L-Phe on the four mutants was also assessed by changes in IP_1_ production upon ligand stimulation. The addition of 5 mM L-Phe significantly elevated the level of IP_1_ accumulation in cells transfected with WT CaSR at 2.0 and 3.0 mM [Ca^2+^]_o_, resulting in a left-shifted [Ca^2+^]_o_-IP_1_ concentration response curve (EC_50_ = 2.0±0.1 mM, p<0.05). In the case of the mutant P221Q, the EC_50_ was reduced from 6.1±0.3 mM to 4.5±0.4 mM in the presence of L-Phe (p<0.05). On the contrary, L-Phe failed to reduce the EC_50_ of L173P significantly (9.0±0.9 mM). The EC_50_s of the IP_1_ responses for the two receptors with activating mutations, L173F and P221L, were not altered by L-Phe either (Figure 5, Table 2). Thus results from the IP_1_ experiments were consistent with the [Ca^2+^]_i_ readouts in terms of EC_50_.

L-Phe also had an impact on the [Ca^2+^]_o_-triggered ERK_1/2_ activities of WT and mutant CaSRs in CaSR transfected cells. As shown in Figure 6 and Table 2, the amount of phosphorylated ERK_1/2_ was increased significantly by L-Phe at 2.0 mM [Ca^2+^]_o_ in the WT CaSR. The ERK_1/2_ activity of the WT CaSR in the presence of L-Phe reached a maximum at 4.0 mM [Ca^2+^]_o_. Quantitative analysis of the immunoblots suggests that L-Phe shifted the sigmoidal curves to the left producing a lower EC_50_s for [Ca^2+^]_o_-evoked ERK activity. In accordance with the [Ca^2+^]_i_ responses and the IP_1_ accumulation readouts, the addition of 5 mM L-Phe shifted the [Ca^2+^]_o_-stimulated p-ERK_1/2_ concentration response curve of mutant P221Q to the left, resulting in a decrease in the EC_50_ from 11.9±0.3 mM to 9.5±0.4 mM (p<0.05), but had little influence on the activity of mutant L173P (Figure 6, Table 2). On the other hand, the maximum ERK_1/2_ activity in cells transfected with the L173F mutant was achieved at 3.0 mM [Ca^2+^]_o_ with 5.0 mM L-Phe, significantly shifting the [Ca^2+^]_o_-triggered [Ca^2+^]_i_ concentration response curve to the left. For the mutant P221L, the addition of L-Phe failed to activate the ERK_1/2_ signaling at lower levels of [Ca^2+^]_o_, but the maximal p-ERK_1/2_ activity was enhanced ∼15% in the presence of L-Phe. The differences of the ERK activity results compared with the IP_1_ readouts suggested that L-Phe may differentially change the heterotropic cooperativity via multiple signaling pathways.

#### CaSR mutants exhibit different sensitivities to L-Phe

**Figure 7:**
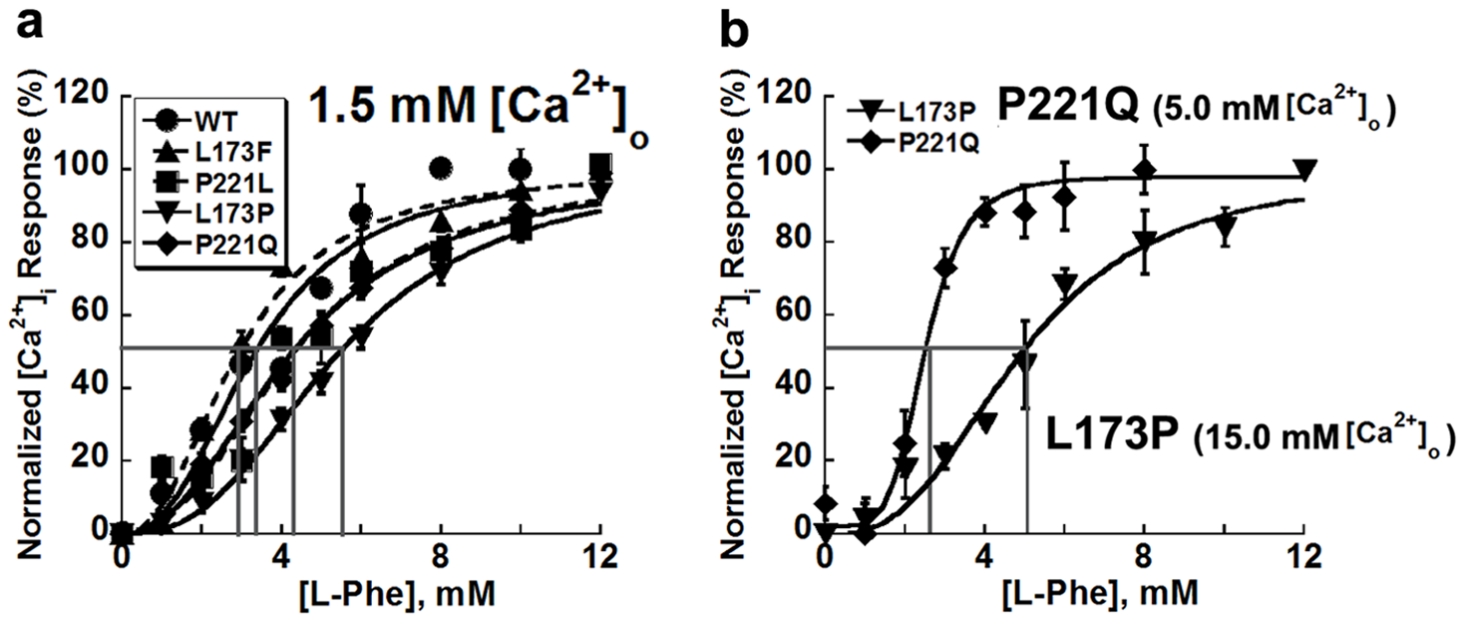
Sensitivity of various CaSR mutants to L-Phe in HEK293 cells. a. HEK-293 cells transfected with CaSR or its mutants were loaded with Fura-2 AM for 15 min. The intracellular Ca^2+^ level was assessed by monitoring emission at 510 nm with excitation alternately at 340 or 380 nm using fluorescence microscopy as above. Each experiment started with 1.5 mM mM Ca^2+^ followed by stepwise increases in the level of L-Phe (up to 12.0 mM) while the [Ca^2+^]_o_ was maintained at 1.5 mM. b. The sensitivity of P221Q and L173P to L-Phe were compared at 5.0 mM [Ca^2+^]_o_ or 15.0 mM [Ca^2+^]_o,_ respectively.

To determine whether or not the distinctive effects shown by L-Phe on different mutants are due to changes in their apparent affinities for this amino acid, concentration responses for L-Phe at a physiological level of 1.5 mM [Ca^2+^]_o_ were first compared among cells transfected with different CaSR mutants. Figure 7 shows that at 1.5 mM [Ca^2+^]_o_, the loss-of-function mutant L173P exhibited a lower sensitivity (EC_50_ = 5.5±0.3 mM) to L-Phe compared to WT and the other mutants (all EC_50_s<5.0 mM). Since the influence of L-Phe might, to some extent, depend on the extracellular calcium concentration, the apparent affinities of L-Phe for the two loss-of-function mutants were further analyzed at levels of [Ca^2+^]_o_ equal to their respective EC_50_s for [Ca^2+^]_o_, specifically 15.0 mM [Ca^2+^]_o_ for L173P and 5.0 mM [Ca^2+^]_o_ for P221Q. P221Q exhibited a reduced EC_50_ for the L-Phe response (EC_50_ = 2.5±0.2 mM, p<0.05) at 5.0 mM [Ca^2+^]_o_ compared with EC_50_ for L-Phe measured at 1.5 mM [Ca^2+^]_o_ (EC_50_ = 4.5±0.4 mM), while L173P retained a similar sensitivity to L-Phe regardless of the extracellular calcium concentration (EC_50_ = 4.9±0.5 mM, p>0.05) (Figure 7, Table 3).

**Table 3:** Summary of EC_50_s of concentration-response curves for L-Phe at different [Ca^2+^]_o_.

Mutants	EC_50_ (at 1.5 mM [Ca^2+^]_o_)
**WT**	3.7±0.4
**L173F**	3.2±0.2
**P221L**	4.3±0.3
**L173P**	5.5±0.3^#^ (4.9±0.5)
**P221Q**	4.5±0.4 (2.5±0.2*)

The [Ca^2+^]_i_ responses of HEK-293 cells transfected with CaSR or its mutants upon stepwise increases of L-Phe in the presence of 1.5 mM [Ca^2+^]_o_ were recorded. For L173P and P221Q, the L-Phe induced intracellular change were also measured in the presence of a level of high [Ca^2+^]_o_ corresponding to their EC_50_ values for the [Ca^2+^]_o_, specifically 15.0 mM [Ca^2+^]_o_ for mutant L173P and 5.0 mM for P221Q. The results are shown in the brackets. The EC_50_s were calculated from the concentration-response curves fitted using the Hill equation. ^#^ indicates significance with respect to wild type CaSR, p<0.05 (ANOVA, Dunnett test); ^*^ indicates significance with respect to the corresponding mutants in the presence of 1.5 mM [Ca^2+^]_o_, p<0.05 (two-way ANOVA).

#### Computational simulations reveal different dynamic behaviors of the four mutants

**Figure 8:**
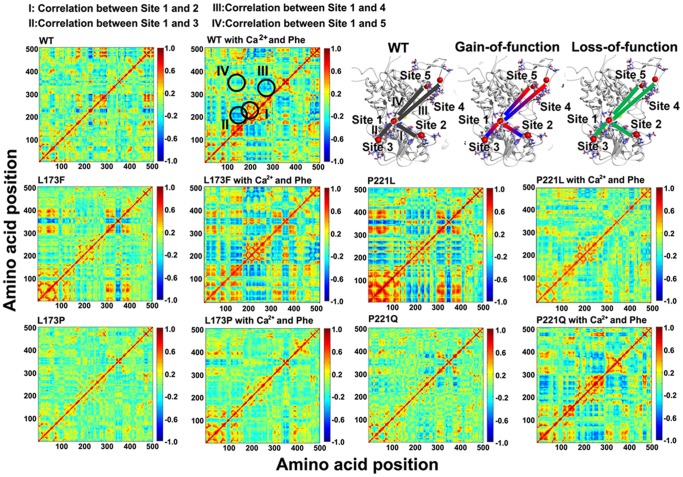
Correlation map of the modeled WT and mutant CaSR ECD structures. The correlation maps are depicted based on molecular dynamic (MD) simulations of the WT and mutant CaSR ECD structures in the apo form or in the presence of Ca^2+^ and L-Phe. The X axis and Y axis are residue numbers of the CaSR ECD sequence minus twenty four. The strongest negative correlation is given the value -1, while the strongest positive correlation is defined as +1. Residues that have the strongest negative correlated motions are shown in blue, while those involved in positive correlated motions are shown in red. Green indicates no apparent correlated motion between the two residues. The correlated motions between calcium binding site 1 and the other calcium binding sites are mapped onto the CaSR ECD models as shown in the top right panel.

The correlated motions in Figure 8 illustrate the molecular dynamic motions of WT CaSR and its mutants obtained from computational simulations. In the modeled WT structure, Site 1 has strong correlated motions with sites 2, 3 and 4, shown in blue (negative correlated motions between a pair of residues signify motions in opposite direction) and red (positive correlations, which are movements in the same direction) (Figure 8, top two panels). We also have reported in our earlier work that the presence of both L-Phe and Ca^2+^ in site 1 produces greater correlated motions among the Ca^2+^-binding sites compared with L-Phe alone in site 1 [12]. Interestingly, the loss-of-function mutations, L173P and P221Q, exhibit less correlated motions compared to the WT CaSR as demonstrated by a decrease in the negative (blue) and/or positive correlated motions (Figure 8, bottom panel). On the other hand, the correlated motions of the gain-of-function mutants, L173F and P221L, are dramatically increased in regions similar to those of the WT correlated motions in the presence of L-Phe, indicating an enhancement of correlated motions between the respective residues in these two mutants (Figure 8, middle panel). A closer analysis of the correlated motions of L173F and P221L reveals that the negative correlations between Site 1 and Site 3 and between Site 1 and Site 2 observed in the WT correlation map become positive correlations in the gain-of-function mutants indicating that the mutations might have profound impacts on the dynamic properties of the CaSR-ECD. Especially for P221L, strong negative correlations between lobe 1 and lobe 2 are observed as indicated by the abundant blue area between residues 200–300 and residues 24–170. These results indicate that residues Leu^173^ and Pro^221^ play pivotal roles in modulating the molecular connectivity between the binding sites for [Ca^2+^]_o_. In order to examine the possibility that the effects of inactivating mutations of the CaSR can be cancelled by those of activating mutations through molecular correlated motions, two double mutations, L173F/P221Q and L173P/P221L have been analyzed. We found that L173F/P221Q behaved like WT CaSR, thereby exhibiting a “cancelling” effect (Figure S1a), while L173P/P221L exhibited oscillation patterns similar to those of the L173P mutant, suggesting that the activating mutant P221L cannot overcome the inactivating effect of L173P (Figure S1b). These results suggest that mutations close to calcium binding Site 1 may play key roles in modulating correlated motions of the CaSR.

The docking of L-Phe to CaSR mutant P221Q engendered a more correlated molecular dynamics features similar to WT CaSR ECD, suggesting that the receptor is converted into an active conformation. Intriguingly, the other inactive mutant L173P did not exhibited dramatic alterations in its correlated motion in response to L-Phe compared to P221Q. This result suggests that the difference in the influence of L-Phe on potentiating the activity of the CaSR mutants may rely on the intrinsic molecular dynamic connectivity that is disrupted by mutant L173P. It is worth noting that the activating mutant L173F also showed enhanced correlated motions, which could be an explanation for the left shift curve of the ERK activity. The addition of L-Phe was unable to make the profile of mutant P221L into a more dramatically active state, which correlated well with the *in vitro* studies, suggesting the mutation is already in a “fully active” state upon the binding with Ca^2+^ similar as WT CaSR with both Ca^2+^ and L-Phe.

**Figure 9:**
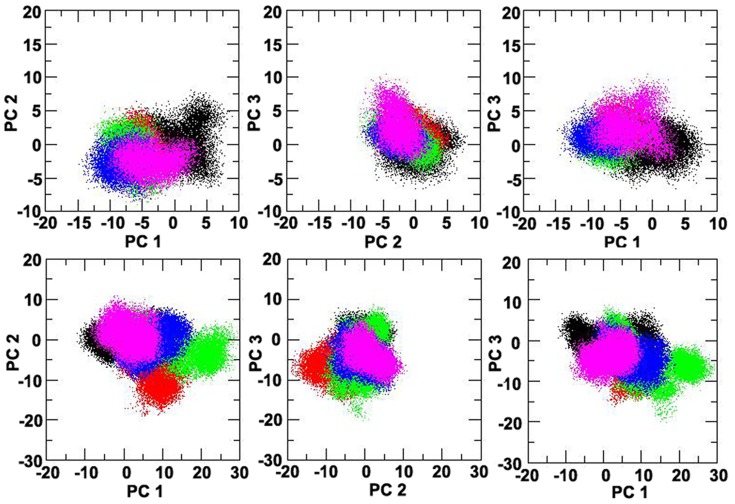
Principal component analysis (PCA) of the CaSR ECDs. The trajectories of the molecular dynamics simulations were analyzed using principal component analysis (PCA), which separates out the motions of the CaSR ECD into principal modes ranked according to their relative contributions. The first three principal modes were included in the present study to analyze the four CaSR mutants and the wild type: WT (black), L173F (green), presence P221L (magenta), L173P (red), P221Q (blue).

Principal component analysis (PCA) separates out the protein motions into principal modes ranked according to their relative contributions [27]. The analysis was done on the trajectories of the four mutations and the WT CaSR from the molecular dynamics simulations in order to predict the effect of the mutations on the response of L-Phe and Ca^2+^ at the atomic level (Figure 9).

Projection of the trajectories of the CaSR mutants in both the apo forms (upper panel) and the holo forms (lower panel) onto the first three modes that accounted for the majority of the total fluctuations is shown in Figure 9. In the absence of Ca^2+^ and L-Phe, the conformations sampled by the CaSR mutants are similar to that of the WT CaSR. However, in the L-Phe and Ca^2+^-loaded forms, the conformations of the CaSR mutants L173P and L173F are distinguishable from the other mutants and WT CaSR (Figure 9). The results suggest that Ca^2+^ and L-Phe might shift the population of conformational ensembles of mutant L173P and L173F in a different way than in WT CaSR or in the other two mutants, supporting the essential role for the residue Leu^173^ in the molecular connectivity underlying the Ca^2+^ - and Phe-mediated functional cooperativity of CaSR.

## Discussion

### Loss-of-function mutations impair the functional cooperativity of the CaSR

The CaSR hinge region is considered to be crucial for the sensing of agonists (e.g., polyvalent cations and amino acids) [19], [28]. Mutations near Ca^2+^-binding site 1 are hypothesized to influence the functional cooperativity of the entire receptor. *In vitro* studies showed that the L173P and P221Q mutants produce a receptor with reduced positive homotropic cooperativity and impaired capability to sense [Ca^2+^]_o_, while the L173F and P221L mutants enhance the sensitivity of the CaSR to [Ca^2+^]_o_ compared to the WT receptor, but barely change the cooperativity of the receptor. The results with the loss-of-function mutants indicate that Leu^173^ and Pro^221^ are important in maintaining the positive homotropic cooperativity among the receptor's calcium-binding sites. Conversely, the gain-of-function mutants show that these residues also are important for restraining the receptor from assuming its most active form (e.g., that seen in the WT CaSR in the presence of both high [Ca^2+^]_o_ and L-Phe). Thus unlike mutations of residues that cause only gain- or loss-of-function, Leu^173^ and Pro^221^ serve dual functions, perhaps reflecting their key locations in the cleft between the receptor's two lobes and their proximities to the binding sites for both [Ca^2+^]_o_ and L-Phe.

[Ca^2+^]_i_ oscillations have been postulated to be the result of complex responses from multiple signaling pathways. The [Ca^2+^]_o_-triggered [Ca^2+^]_i_ oscillation pattern is believed to be modulated by the activity of phosphoinositide pathway [29] as well as a negative feedback loop involving the inhibitory effect of protein kinase C (PKC) on IP_3_ production [30]. Downstream intracellular signaling responses, such as the production of IP_1_ and the activity of ERK_1/2_, which will be discussed later, can be reflected in the oscillation patterns associated with the different disease-related mutations. Although most research has focused on intracellular calcium mobilization in HEK293 cells heterologously expressing the CaSR, CaSR-induced [Ca^2+^]_i_ oscillations have also been found in cells with endogenously expressed CaSR, for instance in parathyroid cells [31], bovine anterior pituitary cells [32], opossum kidney (OK) cells [33] and medullary thyroid carcinoma cells [34]. Oscillations in [Ca^2+^]_i_ modulate not only the rate of parathyroid hormone (PTH) secretion, but also gene expression and other processes [35]–[38]. The pattern of [Ca^2+^]_i_ oscillations is thus one of the most important signatures reflecting the state of CaSR activity. In the present study, we showed that the inactive mutants required higher [Ca^2+^]_o_ to trigger intracellular [Ca^2+^]_i_ oscillations compared to the WT CaSR, while the active mutants needed lower [Ca^2+^]_o_ to initiate oscillations. The oscillation frequency of the inactive mutants remained similar to that of the WT CaSR, but higher [Ca^2+^]_i_ oscillations frequencies were observed in HEK293 cells transfected with gain-of-function mutations. As [Ca^2+^]_o_ is increased, the [Ca^2+^]_i_ oscillations cease and reach a plateau in CaSR-transfected cells as the desensitization process occurs and/or there is depletion of [Ca^2+^]_i_ stores. Although the loss-of-function mutation L173P still oscillated at 30.0 mM [Ca^2+^]_o_, considering the elevated and broad range of its sensitivity to [Ca^2+^]_o_, it is possible that a plateau would be reached at even higher levels of [Ca^2+^]_o_.

Our studies of CaSR-mediated accumulation IP_1_ (a metabolite of IP_3_) and activation of the ERK_1/2_ pathways in HEK293 cells transfected with the various CaSR mutants conveyed two messages: Firstly, the mutations caused changes in the patterns of [Ca^2+^]_i_ oscillations that at least partially involve activation of the Gα_q/11_ pathway. The changes in the accumulation of IP_1_ upon stimulation with various concentrations of [Ca^2+^]_o_ correlated well with [Ca^2+^]_i_ changes. Secondly, high [Ca^2+^]_o_-evoked increases in ERK_1/2_ activities of WT-CaSR and its four mutants were in accordance with the accumulation of IP_1_. Stimulation of the ERK_1/2_ activity in CaSR-transfected HEK293 cells has been reported to involve PKC-mediated as well as a PTX-sensitive, tyrosine kinase-dependent pathways [39]. Although it is believed that the carboxyl terminus of the protein is involved in the activation of MAPK signaling by interacting with the scaffold protein, filamin A [40], [41], other data showed that that alterations in the extracellular domain can also affect the ERK cascade, which is in agreement with previous studies [42]. Since all the mutations studied here were expressed at similar levels on the cell membrane (Figure 1b), these downstream signaling changes may at least partially be contributed by the disruption of cooperativity among different calcium binding sites introduced by different mutations. On the other hand, the gain-of-function mutations may increase the stability the receptor as suggested by other studies [43], or they possibly induce more correlated motions among the calcium-binding sites as suggested by the dynamic cross-correlation map.

### L-Phe-induced heterotropic cooperativity rescues activity of the CaSR

In the current study, we showed that L-Phe could affect one or two of the three parameters depicting the [Ca^2+^]_o_ triggered [Ca^2+^]_i_ oscillation patterns in all of the mutants as measured in individual cellular responses (e.g., [Ca^2+^]_i_ oscillation starting points, ending points and [Ca^2+^]_i_ oscillation frequencies). The alterations in the oscillation patterns after the introduction of the allosteric activator could be the result of the activation of signaling downstream of G_α12/13_ or a combined effect with the positive heterotropic cooperativity between the multiple Ca^2+^ binding sites induced by L-Phe. The interaction between CaSR and L-Phe has been reported to modulate the G_α12/13_-RhoA pathway [44]; however, L-Phe could also potentially enhance [Ca^2+^]_o_-induced alterations in intracellular signaling pathways through G_q/11_ pathway as reflected by the IP_1_ accumulation assay.

The present study also confirms that the ERK signaling pathway in CaSR-transfected HEK293 cells can be modulated not only by [Ca^2+^]_o_ but also by the positive allosteric modulator, L-Phe [45]. It should be noted that the ability of L-Phe to enhance the intracellular responses of the various signaling pathways varied between different mutations. L-Phe enhanced the maximum responses of the ERK signaling pathway to a greater extent than it altered the apparent affinities in the two gain-of-function mutants, although it barely showed any influence on the [Ca^2+^]_o_-triggered [Ca^2+^]_i_ and IP_1_ responses for these mutants, which may explain, in part, the dramatic changes in correlated motions from the computational simulation results using molecular dynamics. As a signaling messenger further downstream than either IP_1_ or [Ca^2+^]_i_, the phosphorylation of ERK_1/2_ may not merely reflect activation of the G_q/11_ pathway, but could be contributed to by other G-proteins (e.g., G_i_). In agreement with the in silico results, L-Phe exhibited less effect on the regulation of the ERK_1/2_ pathway in mutant L173P. The predicted L-Phe-binding site has been reported to be located near the hinge region between lobe 1 and lobe 2 and to involve residues S169A/S170A/S171A [46]. Leu173 is located within 5 Å of this region. The Leu to Pro change could potentially generate additional steric effects on the closure of lobe 1 and lobe 2 so that addition of exogenous L-Phe has relatively little impact on the CaSR's response to changes in [Ca^2+^]_o_. Thus, the hetero-cooperativity between the allosteric modulator and calcium binding sites could be disturbed, resulting in the lack of a left-shifted concentration-response curve in response to [L-Phe].

### Disease-associated mutations influence the molecular connectivity between the Ca^2+^-binding sites of the CaSR as revealed by molecular dynamic simulations

**Figure 10:**
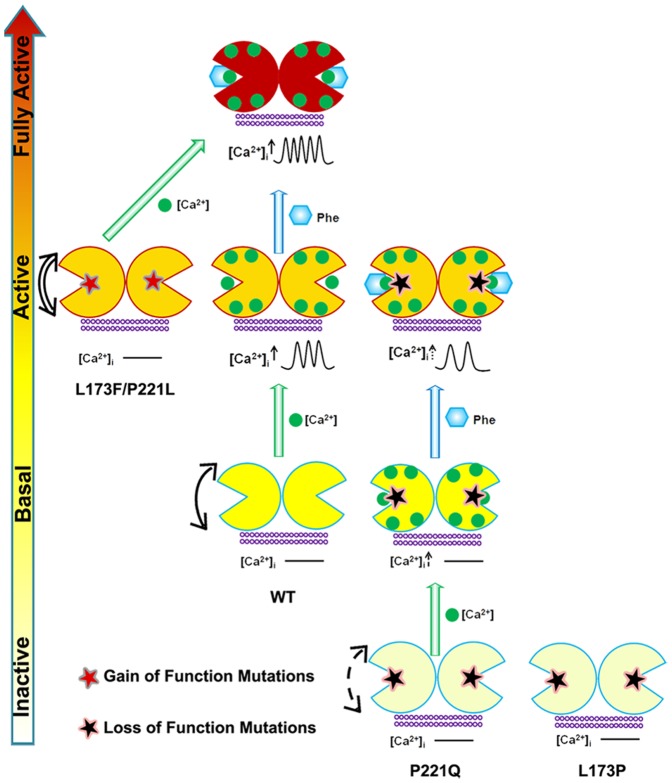
Schematic representation of the mechanisms underlying the effects of the mutations on the CaSR and the modulation of receptor activity by extracellular Ca^2+^ and L-Phe. Ca^2+^ and L-Phe modulate the activity as well as the cooperativity of CaSR (the color changes of the receptor from white to red indicates an increase in functional activity). Elevating [Ca^2+^]_o_, e.g., to 3.0 mM, is proposed to change the basal CaSR status into an active form in a positive homotropic cooperative manner and further trigger [Ca^2+^]_i_ oscillations. L-Phe binds to the hinge region between lobe 1 and lobe 2, modulating the receptor together with Ca^2+^ in a positive heterotropic cooperative way. This could potentiate conversion of the receptor to a “fully active” form associated with a higher frequency of [Ca^2+^]_i_ oscillations and a left-shifted EC_50_. Loss-of-function CaSR mutants (indicated by white color) could cause a disruption of the cooperativity among the various Ca^2+^-binding sites (dashed arrows). [Ca^2+^]_o_ at 3.0 mM does not trigger [Ca^2+^]_i_ oscillations in the mutant CaSRs. The impaired receptor function and the cross-talk between Ca^2+^-binding sites can be at least partially rescued for some mutants by L-Phe (e.g.P221Q). However, if the mutation interferes the interaction between CaSR and L-Phe, the function of the receptor may not be fully recovered (e.g.L173P). CaSR gain-of-function mutants (left) exhibit enhanced correlated motions (double line arrows) and their activity is not further potentiated by L-Phe, potentially due to a ceiling effect.

Based on all of the *in vitro* results and the *in silico* simulations, we propose a model to illustrate the possible mechanism by which the disease-associated mutations affect the function of CaSR and how their activities can be modulated by Ca^2+^ and L-Phe through the molecular connectivity that is encoded at the hinge region of the ECD of the protein. As shown in Fig 10, there are four functional states of the CaSR. In the absence of Ca^2+^, WT CaSR is at the basal state can be converted to the active state upon Ca^2+^ binding to site 1 at the hinge region. Subsequent binding of L-Phe at the adjacent hinge region further strengthens molecular connectivity and elevates the receptor to a “fully active” status in the presence of high [Ca^2+^]_o_ by a heterotropic positive cooperative mechanism. Mutations close to the hinge region such as those at Leu^173^ and Pro^221^ affect the overall dynamic correlated motions of the respective mutant receptors and the Ca^2+^-induced homotropic cooperativity, which could further influence the conformation of the receptor. The inactivating mutation L173P impairs the molecular connectivity in the WT CaSR and cannot be rescued by L-Phe. On the other hand, the inactivating mutation P221Q weakens the connectivity but can be partially rescued by binding Phe with a resultant activity similar to the basal activity of the WT CaSR. In contrast, the gain-of-function mutants L173F and P221L may already have been converted to the active state with dynamic properties similar to that of Ca^2+^- loaded WT CaSR. They can be further activated upon binding extracellular Ca^2+^ to reach a state close to the “fully active” state (Phe + [Ca^2+^]_o_ in the WT) that is insensitive to further activation by L-Phe achieving an apparent “ceiling” represented by the maximal achievable activity of the CaSR.

The results from PCA may also provide an explanation as to why L-Phe interacts preferably with the P221Q mutant. L-Phe could not effectively shift the inactive population of conformational ensembles of L173P to an active ensemble of conformations as it did with the other inactivating mutant and WT. However, whether the *in silico* simulations provide a novel method to predict mutational effects on CaSR will ultimately require the kind of structural detail available for the mGluRs, whose structures have been solved. Here, the model structure does not take into consideration the possible interactions between the ECD and the extracellular loops and/or transmembrane domain as well as the intracellular domains. Thus, caution is merited in over-interpreting the *in silico* results. Nevertheless, the predicted structural motions may help to identify the mutations or ligands that produce increased or diminished coupling among specific structural elements within the CaSR ECD. While this type of analysis is limited at the moment to the environment of the orthosteric site, this strategy could facilitate drug design for modulating the functions of disease-related mutations affecting the CaSR, as well as other members of the family C GPCRs.

The distinctive combinatorial effects of L-Phe and [Ca^2+^]_o_ on different mutant CaSRs also suggests that ligand selectivity at the orthosteric site and receptor activity at non-orthosteric sites could both contribute to the function of the receptor. This feature of the adjacent orthosteric binding sites and allosteric regulatory sites that cooperatively regulate the molecular connectivity may be shared by other members of the family C GPCRs [47], [48]. In addition to the Ca^2+^-sensing receptor, [Ca^2+^]_o_ regulates 14 of the other members of the family C G protein-coupled receptors (GPCRs), including the metabotropic glutamate receptors (mGluR), γ-aminobutyric acid GABA_B_ receptors and receptors for pheromones, amino acids and sweet substances [1], [49]–[55]. The observed molecular modulatory mechanism may be shared by other members of the family C GPCRs [47], [48]. For instance, the analyses of multiple point mutations in the taste receptors (T1R1/T1R3) suggested that the combination of the two distinct determinants selectivity at the ligand binding site and receptor activity at allosteric sites may mediate the ligand specificity of T1R1/T1R3 [56].

In conclusion, through analysis of [Ca^2+^]_i_ oscillations and the [Ca^2+^]_o_- and L-Phe triggered downstream signaling changes, functional positive cooperativity of CaSR was revealed to be disrupted in the identified FHH-associated CaSR mutations, L173P and P221Q, but not in the ADHH related mutations, L173F and P221L. The addition of L-Phe rescued the function of P221Q by inducing heterotropic positive cooperativity. The distinctive correlated motions of the gain-of-function mutations, on the one hand, and the loss-of-function mutations, on the other, and the potential utility revealed by the molecular dynamic simulation in providing insights into the mechanism for the disease-associated functional alterations in the receptor may also apply to other GPCR proteins.

## Supporting Information

Figure S1 **Functional studies of the CaSR double mutations in individual HEK293 cells.** The panels show representative oscillation patterns from single cells. HEK-293 cells transfected with L173F/P221Q or L173P/P221L were loaded with Fura-2 AM for 15 min. [Ca^2+^]_i_ was assessed by monitoring emission at 510 nm with excitation alternately at 340 or 380 nm as described in Methods. Each experiment began in Ca^2+^-free Ringer's buffer (10 mM HEPES, 140 mM NaCl, 5 mM KCl, and 1.0 mM MgCl_2_, pH 7.4), followed by stepwise increases in [Ca^2+^]_o_ until [Ca^2+^]_i_ reached a plateau (up to 30 mM [Ca^2+^]_o_). a. Cells were transfected with pEGFP-N1-CaSR L173F/P221Q. b. Cells were transfected with pEGFP-N1-CaSR L173P/P221L.
(DOCX)
