# Supplementary material for: Role of Ca2+ and L-Phe in Regulating Functional Cooperativity of Disease-Associated “Toggle” Calcium-Sensing Receptor Mutations
Source: PLoS One. 2014 Nov 24;9(11):e113622. doi: 10.1371/journal.pone.0113622 (PMC4242666; doi:10.1371/journal.pone.0113622)
Supplement: Figure S1 — Functional studies of the CaSR double mutations in individual HEK293 cells. The panels show representative oscillation patterns from single cells. HEK-293 cells transfected with L173F/P221Q or L173P/P221L were loaded with Fura-2 AM for 15 min. [Ca2+]i was assessed by monitoring emission at 510 nm with excitation alternately at 340 or 380 nm as described in Methods. Each experiment began in Ca2+-free Ringer's buffer (10 mM HEPES, 140 mM NaCl, 5 mM KCl, and 1.0 mM MgCl2, pH 7.4), followed by stepwise increases in [Ca2+]o until [Ca2+]i reached a plateau (up to 30 mM [Ca2+]o). a. Cells were transfected with pEGFP-N1-CaSR L173F/P221Q. b. Cells were transfected with pEGFP-N1-CaSR L173P/P221L. (DOCX) [file pone.0113622.s001.docx]

**
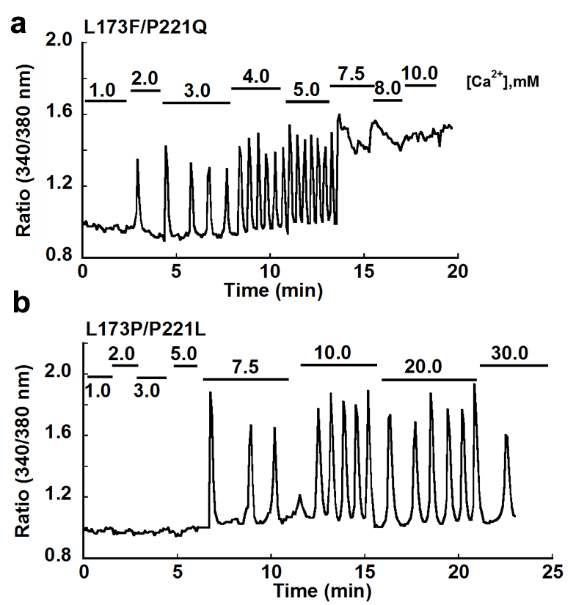
**

**Fig. S1. Functional studies of the CaSR double mutations in individual HEK293 cells.** The panels show representative oscillation patterns from single cells. HEK-293 cells transfected with L173F/P221Q or L173P/P221L were loaded with Fura-2 AM for 15 min. [Ca^2+^]_i_ was assessed by monitoring emission at 510 nm with excitation alternately at 340 or 380 nm as described in Methods. Each experiment began in Ca^2+^-free Ringer’s buffer (10 mM HEPES, 140 mM NaCl, 5 mM KCl, and 1.0 mM MgCl_2_, pH 7.4), followed by stepwise increases in [Ca^2+^]_o_ until [Ca^2+^]_i_ reached a plateau (up to 30 mM [Ca^2+^]_o_). a. Cells were transfected with pEGFP-N1-CaSR L173F/P221Q. b. Cells were transfected with pEGFP-N1-CaSR L173P/P221L.
